# Surface and Subsurface Nitrate Flow Pathways on a Watershed Scale

**DOI:** 10.1100/tsw.2001.336

**Published:** 2001-11-30

**Authors:** C.S.T. Daughtry, T.J. Gish, W.P. Dulaney, C.L. Walthall, K.-J.S. Kung, G.W. McCarty, J. T. Angier, P. Buss

**Affiliations:** USDA-ARS Hydrology and Remote Sensing laboratory, Beltsville, MD 20705, USA

**Keywords:** subsurface flow, nitrogen, preferential flow, topography, remote sensing, yield monitoring, GIS, soil moisture sensors

## Abstract

Determining the interaction and impact of surface runoff and subsurface flow processes on the environment has been hindered by our inability to characterize subsurface soil structures on a watershed scale. Ground penetrating radar (GPR) data were collected and evaluated in determining subsurface hydrology at four small watersheds in Beltsville, MD. The watersheds have similar textures, organic matter contents, and yield distributions. Although the surface slope was greater on one of the watersheds, slope alone could not explain why it also had a nitrate runoff flux that was 18 times greater than the other three watersheds. Only with knowledge of the subsurface hydrology could the surface runoff differences be explained. The subsurface hydrology was developed by combining GPR and surface topography in a geographic information system. Discrete subsurface flow pathways were identified and confirmed with color infrared imagery, real-time soil moisture monitoring, and yield monitoring. The discrete subsurface flow patterns were also useful in understanding observed nitrate levels entering the riparian wetland and first order stream. This study demonstrated the impact that subsurface stratigraphy can have on water and nitrate (NO_3_-N) fluxes exiting agricultural lands, even when soil properties, yield distributions, and climate are similar. Reliable protocols for measuring subsurface fluxes of water and chemicals need to be developed.

## INTRODUCTION

Nitrogen (N) is an essential element for plant growth and is frequently the major limiting nutrient in many agricultural soils. Modern corn production requires large quantities of N because the economic penalties of reduced yields from inadequate N are substantial. Fertilization in excess of a crop's nutritional needs may allow N to move into surface water and groundwater. Farmers must therefore balance the competing goals of supplying enough N to their crops while also minimizing the loss of N to the environment since that represents both a threat to water quality and an economic cost.

In the past decade, there has been an increased awareness associated with the impact of runoff water and sediment on the ecology of surface streams. Runoff is governed by complex interactions of several factors including (1) initial soil water content; (2) soil physical properties, i.e., soil texture and organic matter content; (3) intensity and duration of precipitation events; (4) hydrologic properties of the surface soil, i.e., slope, landscape position, and surface crusting; and (5) subsurface stratigraphy. Although the interactions of the first four of these factors have been studied extensively, the impact of subsurface stratigraphy at the watershed scale is largely unknown. Recent technological advances in ground penetrating radar (GPR), electromagnetic induction (EM), global positioning systems (GPS), and geographic information systems (GIS) should permit detailed analysis of subsurface soil properties and enhance our knowledge of surface and subsurface flow interactions.

EM techniques have been used to estimate relative clay content near the soil surface that might restrict infiltration and influence crop growth[1]. GPR has been used to gather information about the spatial continuity of coarse- and fine-textured beds in sedimentary deposits[2] and to identify gross subsurface stratigraphies[3]. Recently, Dulaney et al.[4] successfully used GPR data to identify water flow along clay lenses in a predominately sandy loam soil. The size, shape, inclination, and spatial correlation of buried soil lenses have been combined with surface topography in a GIS to identify subsurface convergent flow pathways[5]. These flow pathways may alter local hydrology by influencing soil water dynamics above the subsurface pathway.

Our objectives are to (1) assess the relative importance of various soil physical properties on surface runoff at the watershed scale and (2) examine impact of subsurface stratigraphy on surface runoff fluxes of water and nitrate from adjacent watersheds with similar soil characteristics and climate. Traditional methods of determining N budgets for agricultural fields have generally ignored the contributions of subsurface preferential pathways to N loss. In this paper we integrate several state-of-the-art research methodologies to identify subsurface flow pathways and demonstrate their impact on local hydrology and crop yields. Reliable protocols to measure subsurface fluxes of water and N from agricultural fields have yet to be developed.

## EXPERIMENTAL METHODS

### Field Description

Crop and soil characteristics and remotely sensed data were acquired at a 20-ha research field site used for the OPE3 (Optimizing Production Inputs for Economic and Environmental Enhancement) research project. One of the principle objectives of OPE3 is to determine watershed-scale fluxes of water and chemicals from conventional and alternative production systems. The OPE3 field site is located at the USDA Beltsville Agricultural Research Center in Beltsville, MD (39° 02' 00" N, 76° 50' 00" W). Four adjacent watersheds, ranging from 3.4 to 4.1 ha each, were delineated with earthen berms and instrumented to measure surface water runoff fluxes. The soils[6] in each watershed were formed from sandy fluvial deposits with a predominantly sandy loam texture for the first 0.6 m followed by a loam from 0.6 to 0.9 m. Although a continuous clay lens is present throughout the research site, additional fractured lenses may occur sporadically. Each watershed drains into a riparian wetland forest that contains a first-order stream (Fig. 1). Five stream-monitoring stations with V-notch weirs and ultrasonic water level detectors and water samplers were installed in the primary stream channel. About 180 wells with screened segments and piezometers were installed in transects from the stream to the field edges and continuously monitored hydraulic gradients in the groundwater. Surface runoff water and chemical fluxes from each wa-

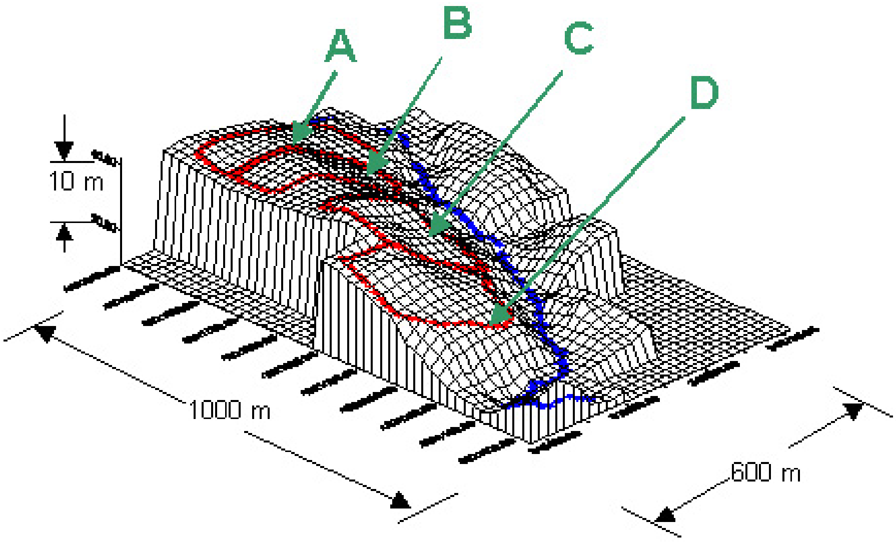

**FIGURE 1.** Topographic relief map of the research site (Z-axis magnified).

tershed were measured with a 1.5-ft H-flume equipped with a flow meter and a water sampler. Two meteorological stations within the OPE3 site and others nearby recorded air temperature, precipitation, and solar radiation. Nutrients and pesticides in the riparian area were monitored periodically in the surface water at the stream weirs and in the subsurface water in the screened wells. Samples of surface runoff water from each watershed were also collected and analyzed for nutrients and pesticides. Nitrate concentrations in each solution sample were determined by ion chromatography. The detection limit for nitrate was 0.5 mg $l^{-1}$.

Characterization of the soils in each watershed was accomplished through the use of soil core sampling and several geophysical techniques. EM was used to locate surface regions of finer-textured soil that might restrict infiltration or influence crop growth[1]. The EM measurements were subsequently verified from a set of 40 soil cores to a depth of 1 m. The depth, size, and orientation of subsurface lenses were determined by analyzing over 40 km of GPR data acquired with a 300 MHz antenna[2,5]. A geostatistical software package (GS, Gamma Design Software, Plainwell, MI) was used to determine the spatial correlation of the depth to the first continuous restricting layer[4]. This program produced omnidirectional experimental semivariograms from point data derived from the digitized traces (i.e., depth to the first continuous restricting layer and its associated geographic coordinates). The semivariograms were model fit using least squares analysis in order to determine kriging parameters—nugget, range, and sill—for subsequent spatial interpolation. Spatial data were stored and manipulated in a GIS.

In March 1999, soil cores were collected to determine the spatial and temporal distribution of a variety of soil chemical and physical properties at 274 sampling locations within OPE3. At each location, soil samples at 0- to 10-cm and 10- to 30-cm depth increments were collected and analyzed for pH, phosphorous, potassium, calcium, organic matter, and clay content. Soil properties were interpolated with ordinary block kriging in GS+.

Corn (*Zea mays* L.) was planted and fertilized using split applications of N as recommended by the University of Maryland. In 1998, the first year of the study, the same management practices (150 kg N/ha) were applied to all watersheds to determine baseline yield distributions. In 1999 and 2000, uniform applications of liquid dairy manure were applied to the soil surface and incorporated prior to planting in watersheds A and C. Because of N loss due to ammonia volatilization, additional N fertilizer was applied (about 50 kg N/ha) as needed according to the Pre-Sidedress Nitrate Test[7] (PSNT). Watersheds B and D received 30 kg N/ha at planting plus additional sidedress N fertilizer according to the PSNT. Leaf area index (LAI), plant height, and development stage were measured and georeferenced with a GPS receiver. Grain yields were measured with a grain combine equipped with a yield monitor and a differential GPS receiver.

Aerial hyperspectral images in the 450- to 850-nm wavelength region were acquired over the OPE3 corn fields on July 16, 1999. The AISA (airborne image spectrometer for different applications) scanner[2] was flown on a small twin-engine aircraft (3Di, Easton, MD). The data were acquired under clear sky conditions near 10:45 a.m. EDT at an altitude of approximately 500 m above ground level. Image data were georectified and corrected to apparent reflectance. The spatial resolution was approximately 1 m. Spectral vegetation indices were computed using 6-nm wide bands centered at 552, 652, and 808 nm, for the green, red, and near infrared (NIR) bands, respectively. Corn LAI[9] was estimated as 0.92 + 0.18 (NIR/Red). Landsat ETM+ and various instruments on the EOS Terra and EO-1 satellites also acquired data over the USDA Beltsville validation site that is centered over OPE3 .

## RESULTS AND DISCUSSION

### Watershed Characteristics

Watershed slopes ranged from 1 to 5%, generally becoming greater as they approached the riparian wetland (Fig. 1 and Table 1). While watersheds A and B have nearly identical surface slopes, watersheds D and C contain significant areas in the larger slope classes. Accordingly, one would expect watershed C to have the greatest runoff, followed by D and then A and B. Although the GPR data indicated the presence of fragmented clay lenses throughout the soil profiles, the first continuous clay lens (restricting layer) ranged from 0.9 to 3.5 m deep across all watersheds (Fig. 2).

The physical properties of the soils in the four watersheds are similar and exhibited significant spatial structures that were well described by the semivarigram models[6]. The soil data were kriged to develop maps that represent the spatial distribution of each soil characteristic (Fig. 3). For example, watersheds A and D had nearly identical distributions of organic matter while watersheds B and C contained about 1% more organic matter than watersheds A and D. Clay contents were similar between watersheds B and C while watershed A had slightly higher clay content and watershed D had slightly lower clay content. The spatial

**TABLE 1**
**Percent of Each Watershed in Various Slope Classes**

| Watershed | Area (ha) | Surface Slope Class | | | | | |
|---|---|---|---|---|---|---|---|
| | | 0–1% | 1–2% | 2–3% | 3–4% | 4–5% | 5–6% |
| A | 3.6 | 40 | 60 | 0 | 0 | 0 | 0 |
| B | 3.4 | 40 | 53 | 7 | 0 | 0 | 0 |
| C | 4.0 | 0 | 34 | 61 | 3 | 0 | 2 |
| D | 4.1 | 18 | 52 | 30 | 0 | 0 | 0 |

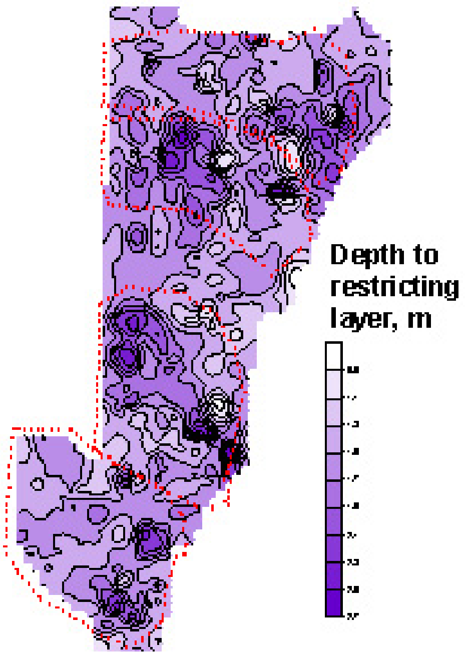

**FIGURE 2.** Depth to the first continuous restricting layer identified by GPR (darker colors indicate greater depth to the subsurface restricting layer).

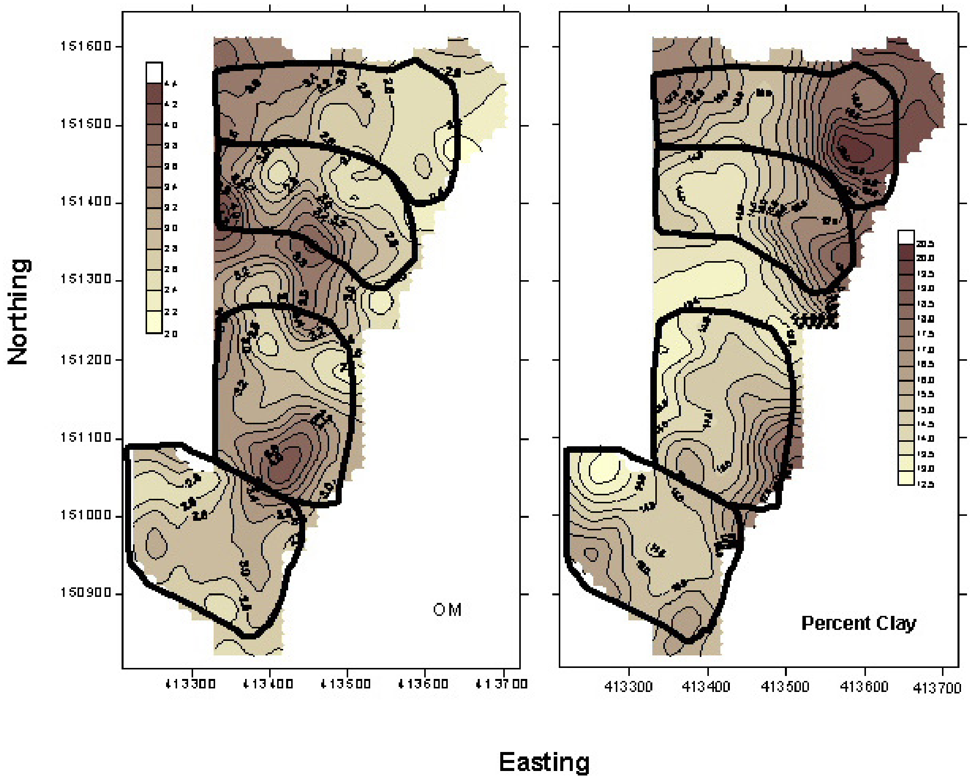

**FIGURE 3.** Spatial distribution of percent organic matter (left) and percent clay content (right) in the surface (0 to 10 cm) soil.

distributions of soil pH, phosphorous, potassium, and calcium were also similar for each watershed (not shown).

Since plants integrate many soil and climatic factors, including water availability, soil fertility, and soil structure, they too can be used to determine watershed similarities. The spatial variability in LAI displayed in the remotely sensed image (Fig. 4) was due to variations in plant growth associated with differences in available soil moisture in a

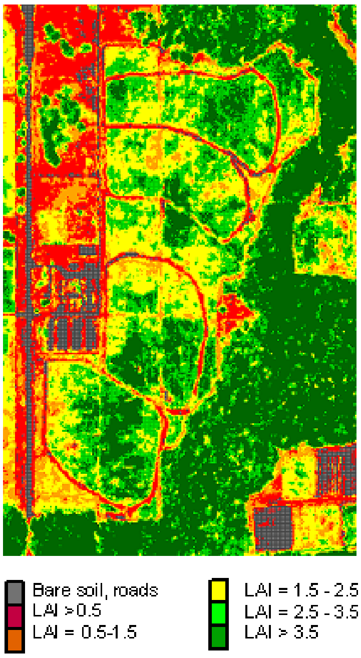

**FIGURE 4.** Spatial variability in vegetation density derived from analysis of imaging spectrometer data acquired on July 16, 1999.

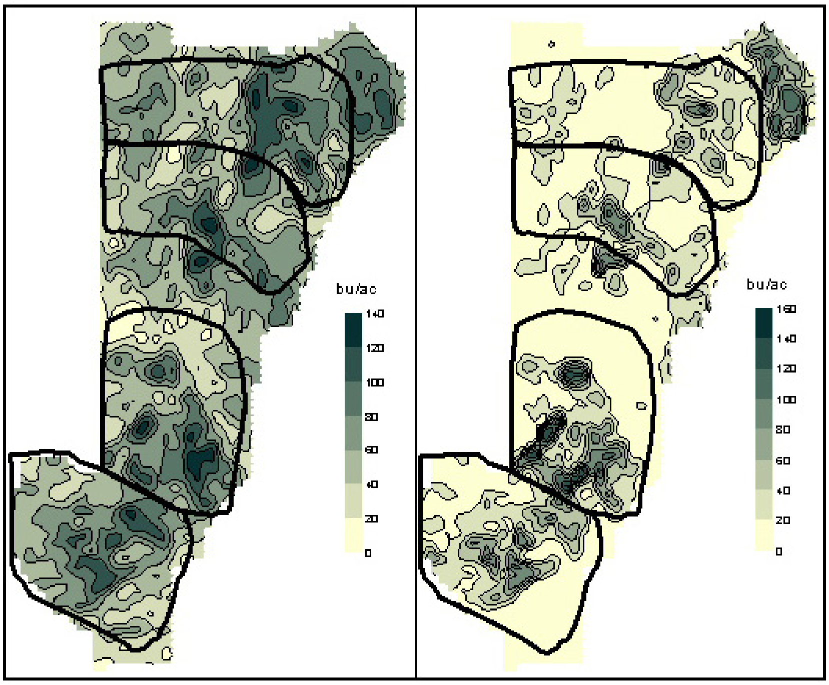

**FIGURE 5.** Spatial distribution of normalized corn grain yields. Darker colors indicate higher yields.

year with below normal rainfall[9]. No significant differences in plant growth were observed among the watersheds. Additionally, although mean grain yield each year varied depending on weather conditions, each watershed had a wide range of grain yields that were normally distributed. There were no significant differences in the spatial distribution of corn grain yields among the watersheds (Fig. 5). Because the four watersheds were so similar in all characteristics except slope, surface runoff was expected to be very similar for all four watersheds.

## Water and Nitrate Runoff Fluxes

Rainfall during the 1999 growing season was below average. No surface runoff was observed until late August 1999, when several major storms including Hurricane Floyd generated significant surface runoff. During this period, surface runoff fluxes were similar for watersheds A, B, and D, but watershed C had a cumulative water flux that was two to three times greater than that of the others (Table 2) even though most of the climatic, soil, and landscape parameters variables indicated that surface runoff values should be similar. Surface runoff from watersheds A, B, and D typically ended within a few hours after precipitation ceased. However, runoff from C often continued for several days or even weeks after a significant rain event. We thoroughly scouted the watersheds and discovered that only watershed C had seepage zones.

Subsurface waters that re-emerge at the soil surface are referred to as seepage. Watersheds with seepage zones are common to agricultural lands, especially those that border riparian wetlands or surface streams[10]. Seepage zones are dynamic, often responding to a single precipitation event, mixing water of differing ages. Depending upon the soil moisture status of the soil profile as well as upon rainfall intensity and duration, the ratio of surface runoff to seepage can change substantially. Seepage zones are also associated with low or decreased downslope water table gradients or decreased permeability[11] (e.g., downgradient shifts from coarse to fine-textured soil).

Although the enhanced surface water flux from watershed C was initially unexpected, the chemical fluxes were even more enlightening. The surface runoff nitrate flux from watershed C was about 18 times greater than other watersheds (Table 2). The main reason for the enhanced nitrate flux was again the seepage zones. As surface runoff water in watershed C became increasingly dominated by seepage flow, the nitrate concentrations in the runoff water would increase dramatically, from 3 to 24 mg $l^{-1}$. On the other hand, late summer surface nitrate concentrations in the other watersheds remained consistent during a runoff event, generally ranging in the 1 to 3 mg $l^{-1}$ range. Gburek[10] also observed that surface runoff from seepage zones had much higher nitrate levels than runoff from areas without seepage.

To determine why seepage zones were observed in only watershed C, the subsurface hydrology of the watersheds had to be understood. First, the location and depth of the subsurface layers that could restrict water flow were identified using GPR. Accordingly, the topography of the subsurface restricting layer was determined by subtracting the depth to the first continuous subsurface restricting layer (Fig. 2) from the surface topography (Fig. 1). Based on the spatial location of the subsurface structures that could intercept and funnel subsurface water, potential subsurface flow pathways were identified using hydrologic routines in Arc/Info (i.e., flowaccumulation and flowdirection). The subsurface channels converged, flowing along the clay lenses, and eventually exited the watershed through discrete channels (Fig. 6). At least one primary subsurface channel exited near the location of the surface runoff flumes in watersheds A, B, and C. (Surface topography of watershed D has not been fully characterized at this time.). Additionally the networks of convergent flow pathways are also spatially correlated the patterns of high corn grain yields (Fig. 6, dashed polygons). The close proximity of high yielding areas to subsurface flow pathways indicated that the plants near the flow pathways were extracting additional water (and nutrients) that were not available to plants in the rest of the field. Thus, knowledge of the subsurface stratigraphy can be extremely important for understanding the spatial dynamics of crop growth and yield when moisture is limiting. The effective drainage areas associated with these primary subsurface channels were 30% for watershed A and 42% for watershed B. However with such small watersheds and only a fraction of the surface area being drained by these primary subsurface flow channels, watersheds A and B never conducted enough subsurface water for the water table to rise from the clay lens to the soil surface. Consequently, most of the infiltrating water remained subsurface when it entered the riparian wetland, and surface runoff was minimal. In contrast, nearly 76% of the area of watershed C was drained by two discrete subsurface flow channels that exited the watershed near the runoff flume (Fig. 6). As the soil profile over these flow channels in watershed C became saturated, seepage zones developed within the watershed. Water from these seepage zones then flowed on the surface exited through the runoff flume and subsequently increased both water and nitrate runoff flux from watershed C.

**TABLE 2**
**Cumulative Surface Water and $NO_3$-N Fluxes Exiting Watersheds during August and September 1999**

| | Water Flux | | $NO_3$-N Flux | |
|---|---|---|---|---|
| Watershed | $m^3$/Watershed | $m^3$/ha | kg/Watershed | kg/ha |
| A | 687 | 191 | 1.27 | 0.35 |
| B | 966 | 284 | 1.86 | 0.55 |
| C | 2678 | 670 | 35.59 | 8.90 |
| D | 840 | 204 | 2.11 | 0.51 |

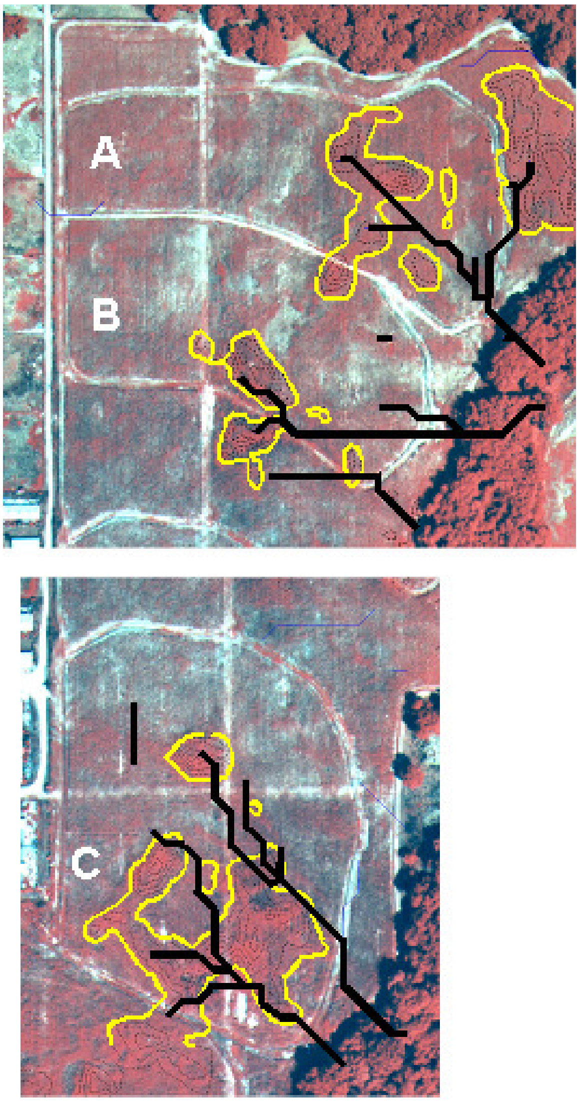

**FIGURE 6.** Color infrared image showing vigorous vegetation (dark red regions), subsurface flow pathways (black lines), and the consistently higher corn producing regions (yellow polygons) for watersheds A, B, and C.

## CONCLUSIONS

Interactions between surface runoff and subsurface hydrology were studied by evaluating subsurface stratigraphy using primarily GPR (Fig. 2) and surface topography (Fig. 1). The four watersheds were similar in all characteristics except slope. Slope alone, however, could not explain the large differences in water and nitrate runoff among the watersheds. Watershed C had almost twice as much surface area draining into discrete subsurface flow pathways as the other watersheds. When these subsurface drainage pathways saturated the soil above them, seepage zones were created which contributed to surface runoff.

This study demonstrated that subsurface hydrology may strongly influence surface runoff even when many soil physical properties are similar. When soil moisture is limited, knowledge of the soil chemical and physical properties normally associated with crop growth contributes little to understanding spatial patterns of crop yields.

## ACKNOWLEDGEMENTS

We gratefully acknowledge the technical assistance of Andrew Russ as well as the support of Dan Shirley and the BARC Farm Operations staff.

## DISCLAIMER

Manufacturer names are provided for information purposes only and do not imply endorsement to the exclusion of others.

| | | |
|---|---|---|
| Received: | July | 20, 2001 |
| Revised: | October | 19, 2001 |
| Accepted: | October | 29, 2001 |
| Published: | November | 30, 2001 |
